# Superior performance of cone beam tomography in detecting a calcaneus fracture

**DOI:** 10.3205/iprs000068

**Published:** 2015-11-05

**Authors:** Christian Lohse, Philip Catala-Lehnen, Marc Regier, Max Heiland

**Affiliations:** 1 Department of Oral and Maxillofacial Surgery, University Medical Center Hamburg-Eppendorf, Hamburg, Germany; 2 LANS Medicum Hamburg, Germany; 3 Diagnostic and Interventional Radiology, University Medical Center Hamburg-Eppendorf, University of Hamburg, Germany

**Keywords:** cone beam computed tomography, computed tomography, calcaneus fracture, trauma

## Abstract

Cone beam computed tomography is a state-of-the-art imaging tool, initially developed for dental and maxillofacial application. With its high resolution and low radiation dose, cone beam tomography has been expanding its application fields, for example, to diagnosis of traumata and fractures in the head and neck area. In this study, we demonstrate superior and satisfactory performance of cone beam tomography for the imaging of a calcaneus fracture in comparison to conventional X-ray and computed tomography.

## Zusammenfassung

Die digitale Volumentomographie ist ein etabliertes Verfahren in der Zahnheilkunde und Mund- Kiefer- und Gesichtschirurgie. Aufgrund der hohen Bildqualität sowie einer hohen Ortsauflösung wird diese Technik mittlerweile ebenfalls in der Traumadiagnostik und im Bereich des Gesichtsschädels erfolgreich eingesetzt. Das Ziel der Arbeit ist eine vergleichende Darstellung zwischen konventioneller Röntgendiagnostik, der Computertomographie und der digitalen Volumentomographie am Beispiel der Beurteilung einer Calcaneusfraktur.

## Introduction

Radiological imaging is an established diagnostic tool for traumata and fractures. At the present, conventional X-ray and computed tomography (CT) are the major tools. However, because of associated radiation risk, the dose of radiation should be kept as low as reasonably achievable [1]. Consequently, the quality and resolution of the imaging can frequently be low. 

**Figure 1:**
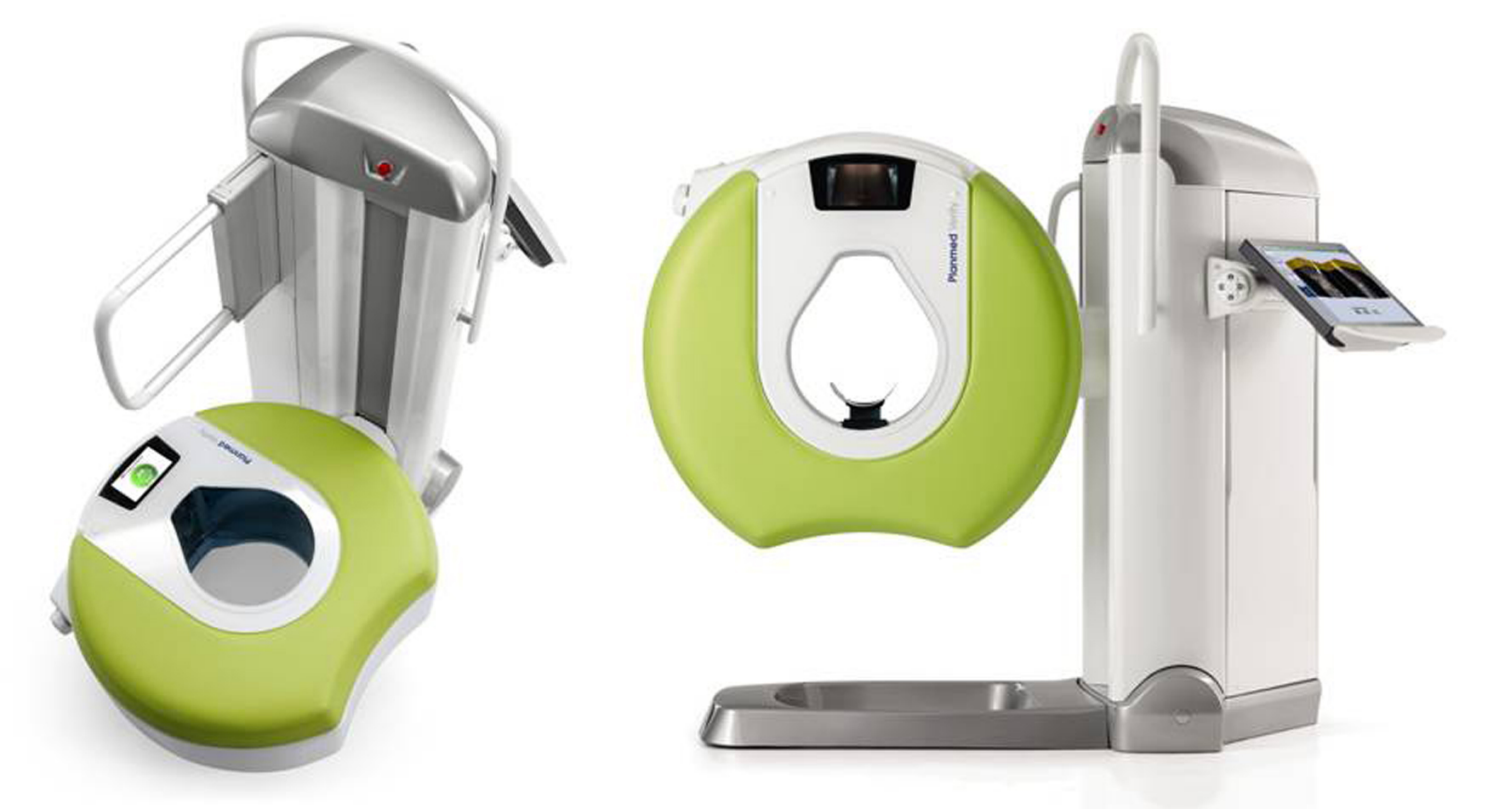
Illustration of the Planmeca Verity CBCT (Planmed Oy, Helsinki)

In 1998, cone beam computed tomography (CBCT) has been developed for imaging the dental and maxillofacial structures. CBCT rotates three-dimensional (3D) radiation beams with corresponding detectors to generate large number of projections from which a 3D reconstruction and volume of the region are calculated in nearly real time. Because of the rotating 3D principle, CBCT demands much low dose of radiation than CT (221 ± 275 mSv vs. 847 ± 313 mSv) [2], [3], [4]. Despite the reduced radiation dose, local resolution of CBCT is substantially higher than that of conventional CT [5]. Furthermore, most CBCT devices are compact and can be operated directly by surgeons (Figure 1 (Fig. 1)). Because of these advantages, the application field of CBCT is being expanding since years. For example, CBCT is becoming a standard imaging tool for dental diagnostic and also traumas in lower jaw and the mid-face conducted by dentists, craniomaxillofacial surgeons and ENTs. Today, more than 47 different types of CBCT devices marketed by 20 companies are available for various applications [6]. 

Fractures of the tarsal bone are frequent, accounting approximately 2% of all skeletal fractures. Among the tarsal fractures, approximately 75% are calcaneus fractures [7]. Due to the complex anatomical structure, precise diagnosis of calcaneus fractures is challenging. Especially for discrete fracture lines without dislocation, a 2D X-ray imaging is often not sufficient and additional CT or magnetic resonance imaging (MRI) are frequently required [8]. Because CBCT has high local resolution, it may provide a superior alternative for detection of calcaneus fractures. Its speeding 3D-reconstruction is also an ideal feature for application in emergency surgery. However, so far, application of CBCT for extremity fractures has not been evaluated. In the present study, we compared CBCT with conventional X-ray and CT regarding their performance in diagnosis of a calcaneus fracture (Table 1 (Tab. 1)). 

## Case description

An accident during cart-ride led to a trauma in the right foot of a 42-year-old healthy male patient. He presented with swelling and pain, and restricted flexibility between talus and calcaneus. 

**Figure 2:**
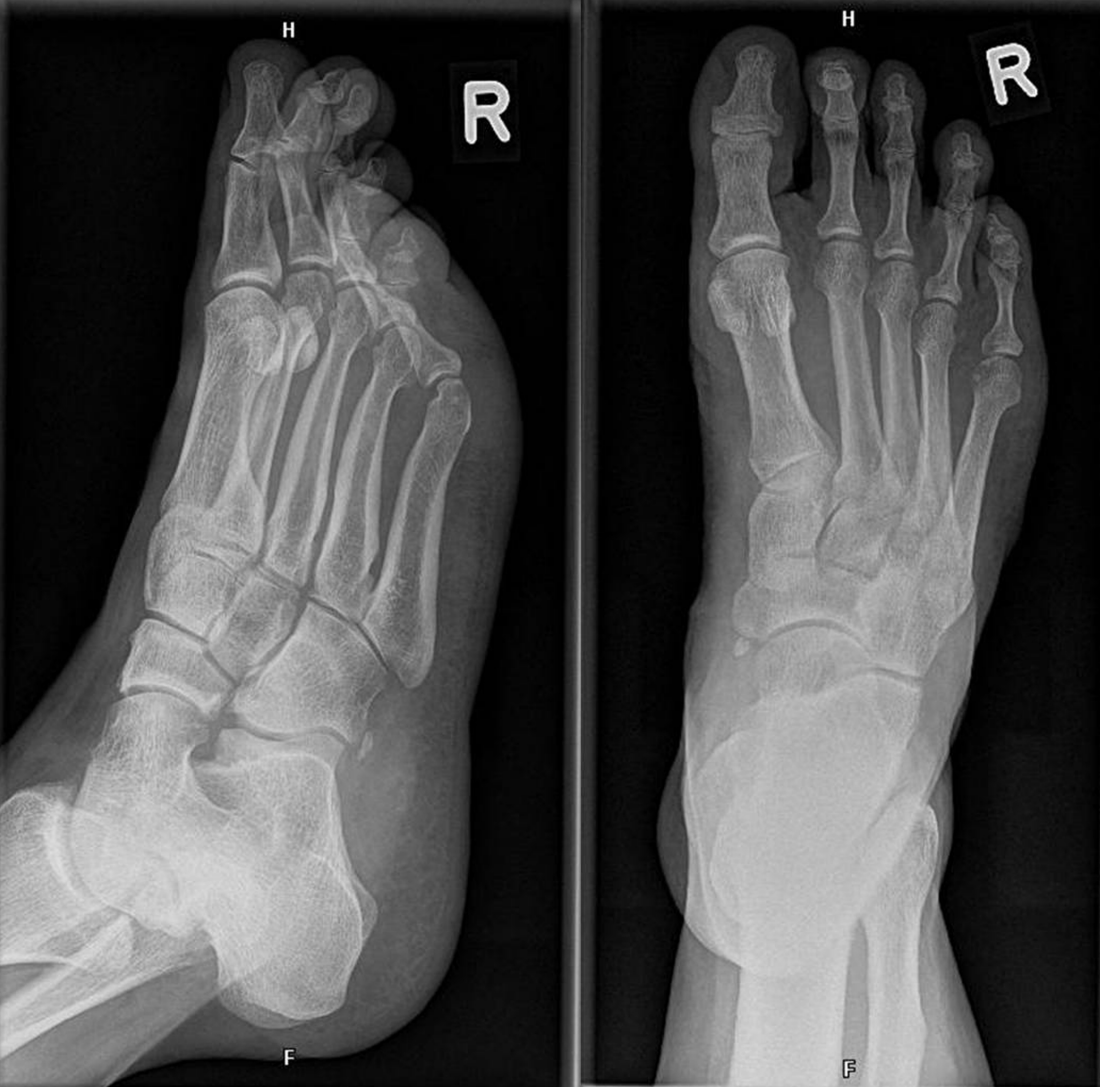
Scan of the calcaneus with a discrete fracture without dislocation of the bone: Conventional X-ray taken at the initial examination

**Figure 3:**
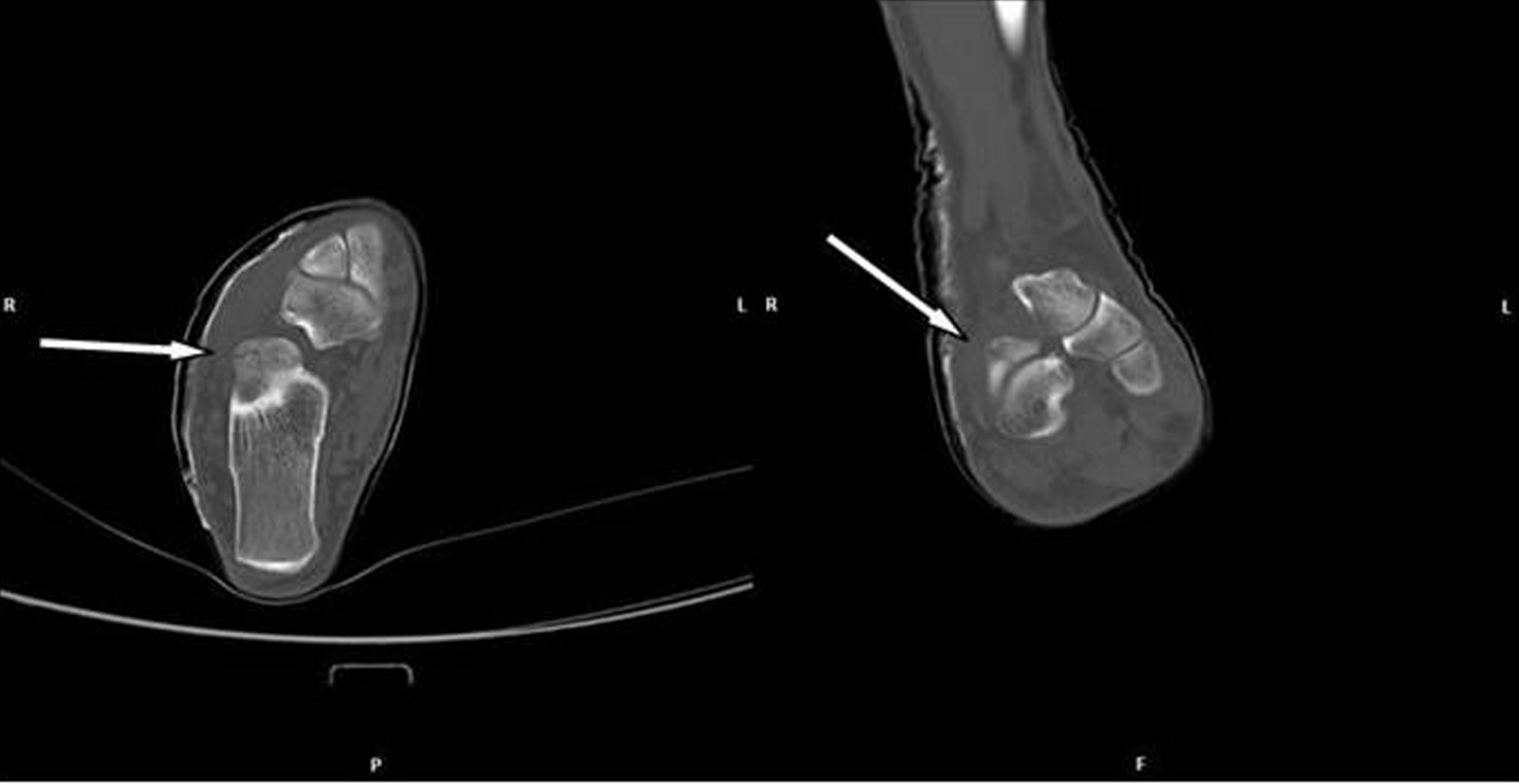
Scan of the calcaneus with a discrete fracture without dislocation of the bone: CT taken at the initial examination. Arrays indicate the fracture lines

The foot with suspected fracture was imaged with conventional X-ray in two plains: axial and mediolateral (Figure 2 (Fig. 2)). However, no disruption of the bone continuity could be seen. Because of persistently pain a MRI scan was performed 10 days after trauma. There was no indication of a ligament rupture but a suspicion of a fracture of the right processus anterior calcanei. A CT scan was therefore conducted which revealed a calcaneus fracture line (Figure 3 (Fig. 3)). 

Since the fracture did not cause dislocation of the calcaneus bone, the patient received a conservative therapy with analgetics (Ibuprofen 600 mg, Ratiopharm GmbH, Ulm, Germany) and a dynamic vacuum orthosis (VACOped). At this moment an immobilization for six weeks was recommended without surgical intervention. 

**Figure 4:**
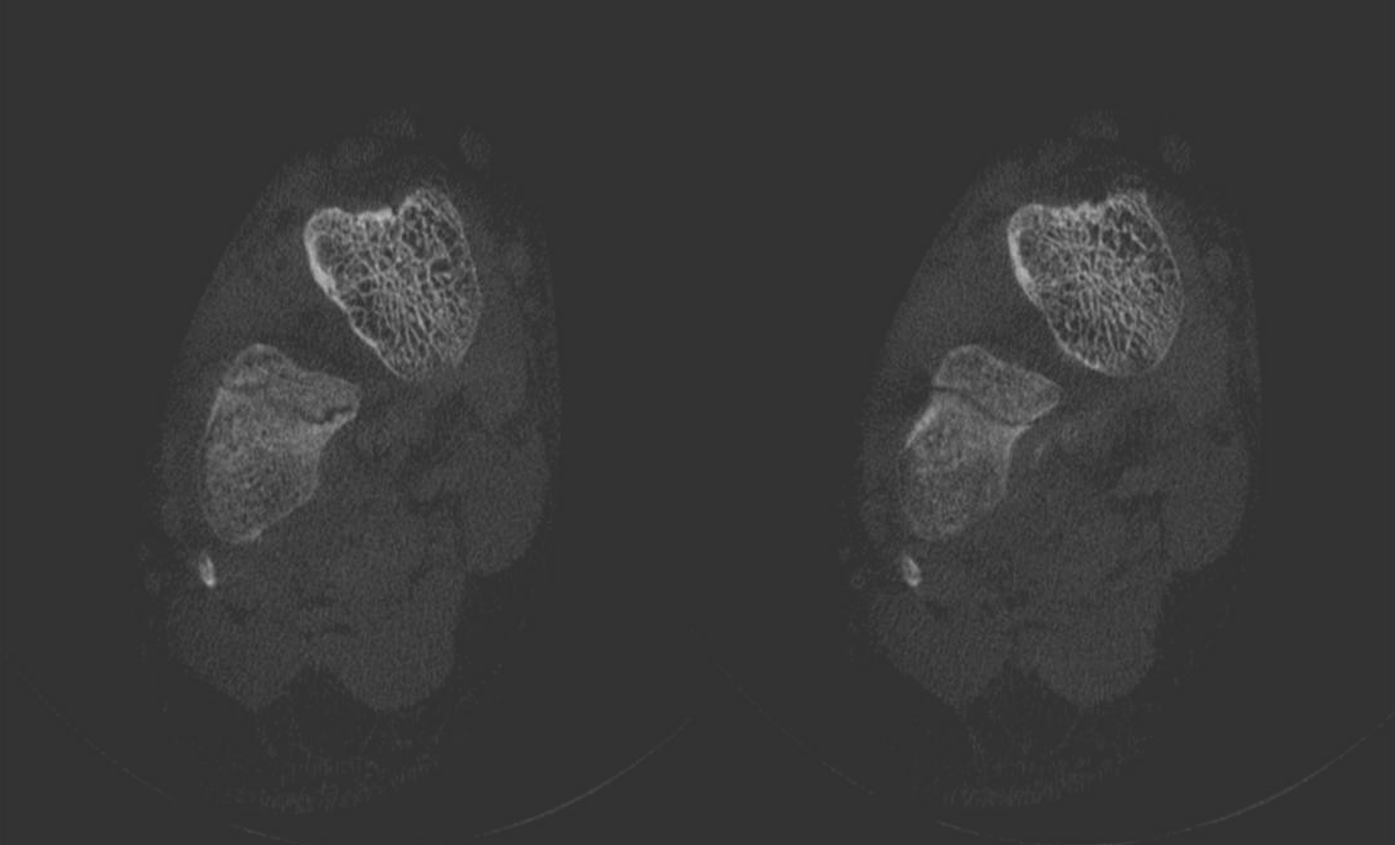
Scans of the calcaneus with a discrete fracture without dislocation of the bone: CBCT taken 6 weeks later at a follow-up examination

Six weeks later, the patient received a control examination for the healing process. To reduce radiation dose, a CBCT was performed. A thin fracture line of the calcaneus was clearly visible (Figure 4 (Fig. 4)). Some sections indicated ossification signs as proof of a healing process (Figure 4 (Fig. 4)). Hence, there was no need for a surgical approach. 

## Discussion

CBCT easily detected a discrete fracture in the complex calcaneus. Comparing with CT, the thin fracture was more distinct in the CBCT scans although the fracture was readily in healing process and therefore should be less visible. Consequently, an even distinct imaging of the fracture could be expected by a CBCT scan at initial diagnosis. 

Beside their high-resolution and low radiation dose, CBCT devices are generally compact and therefore can be installed in even small offices. To date, more than 47 different types of CBCT devices are available for various anatomical regions with varying positioning of the patients [6]. The Planmeca Verity CBCT (Planmed Oy, Helsinki) is especially suitable for imaging extremities. Another important feature is the mobility of this device. Furthermore, most CBCT devices can be operated by surgeons themselves [9]. By contrast, CT usually has to be performed by a radiologist and the scans have to be evaluated by them. Because a CBCT calculates a 3D reconstruction of the scanned region immediately and has an integrated navigation function, it enables intra-operative control and navigation if needed.

While CBCT delivers superior resolution of bone tissues, it is less suitable for imaging of soft tissues. Unlike CT, no contrast reagents can be used for CBCT. Another weakness of CBCT is the lack of normalized density scale [5]. By contrast, CT scans can be evaluated using normalized Hounsfield scale. However, development is in progress toward a standardized radiodensity scale even for CBCT [10].

## Conclusion

Because of its high-resolution scans at low radiation dose, CBCT provides a highly recommended alternative tool also for detecting fractures of extremities. 

## Notes

### Competing interests

This study was supported by SCS Systems Consulting Solutions GmbH, Aschaffenburg, Germany, providing the CBCT device.

## Figures and Tables

**Table 1:**
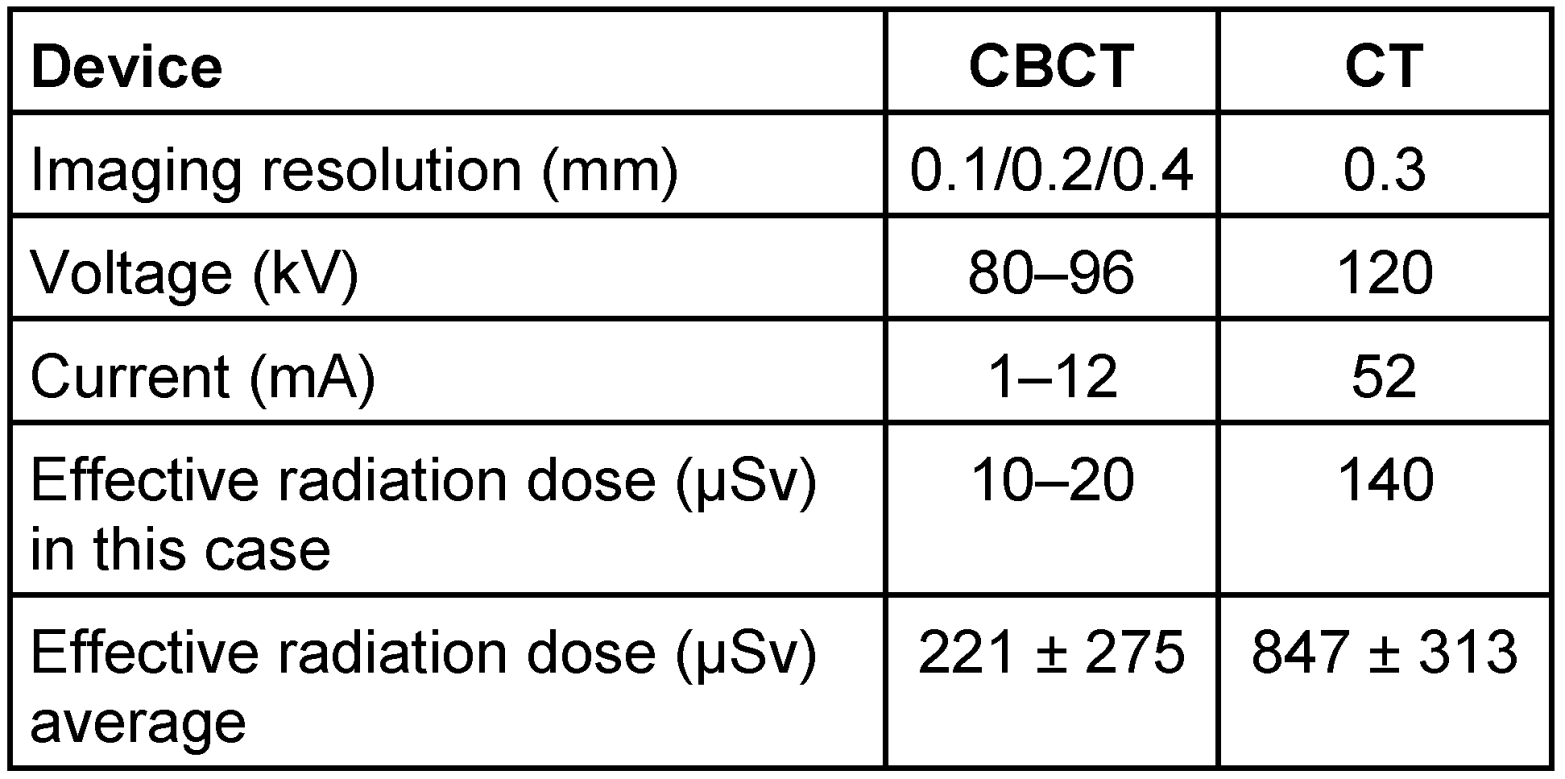
Comparison of CBCT and CT
